# Fine-Tuning of mTOR mRNA and Nucleolin Complexes by SMN

**DOI:** 10.3390/cells10113015

**Published:** 2021-11-04

**Authors:** Francesca Gabanella, Christian Barbato, Marco Fiore, Carla Petrella, Marco de Vincentiis, Antonio Greco, Antonio Minni, Nicoletta Corbi, Claudio Passananti, Maria Grazia Di Certo

**Affiliations:** 1 CNR-Institute of Biochemistry and Cell Biology, Department of Sense Organs, Sapienza University of Rome, Viale del Policlinico, 155-00161 Rome, Italy; christian.barbato@cnr.it (C.B.); marco.fiore@cnr.it (M.F.); carla.petrella@cnr.it (C.P.); 2 CNR-Institute of Molecular Biology and Pathology, Department of Molecular Medicine, Sapienza University of Rome, Viale Regina Elena, 291-00161 Rome, Italy; nicoletta.corbi@cnr.it (N.C.); claudio.passananti@cnr.it (C.P.); 3 Department of Sense Organs, Sapienza University of Rome, Viale del Policlinico, 155-00161 Rome, Italy; marco.devincentiis@uniroma1.it (M.d.V.); antonio.greco@uniroma1.it (A.G.); antonio.minni@uniroma1.it (A.M.)

**Keywords:** SMN, nucleolin, nucleolus, ribosome biogenesis, mTOR, RNA translation, padlock

## Abstract

Increasing evidence points to the Survival Motor Neuron (SMN) protein as a key determinant of translation pathway. Besides its role in RNA processing and sorting, several works support a critical implication of SMN in ribosome biogenesis. We previously showed that SMN binds ribosomal proteins (RPs) as well as their encoding transcripts, ensuring an appropriate level of locally synthesized RPs. SMN impacts the translation machinery in both neural and non-neural cells, in agreement with the concept that SMN is an essential protein in all cell types. Here, we further assessed the relationship between SMN and translation-related factors in immortalized human fibroblasts. We focused on SMN-nucleolin interaction, keeping in mind that nucleolin is an RNA-binding protein, highly abundant within the nucleolus, that exhibits a central role in ribosomes production. Nucleolin may also affects translation network by binding the mammalian target of rapamycin (mTOR) mRNA and promoting its local synthesis. In this regard, for the first time we provided evidence that SMN protein itself associates with mTOR transcript. Collectively, we found that: (1) SMN coexists with nucleolin–mTOR mRNA complexes at subcellular level; (2) SMN deficiency impairs nucleolar compartmentalization of nucleolin, and (3) this event correlates with the nuclear retention of mTOR mRNA. These findings suggest that SMN may regulate not only structural components of translation machinery, but also their upstream regulating factors.

## 1. Introduction

The Survival Motor Neuron (SMN) protein was initially characterized once mutations in its coding gene, SMN1, were linked to motor neurons degeneration in spinal muscular atrophy (SMA) [1,2]. It was subsequently established that SMN is ubiquitously required to sustain fundamental cellular activities in all cell types [3,4,5,6]. The RNA life cycle needs SMN function in key processes such as resolution of R-loops/transcription termination, pre-mRNA splicing, and biogenesis of distinct ribonucleoprotein (RNP) complexes [7,8,9,10]. SMN appears to also be involved in multiple facets of the translation pathway. It promotes intracellular trafficking and local translation of several mRNAs [2,11,12]. The SMN protein may target the mRNA by direct interaction, via its nucleic acid-binding domain, as well as by the association with distinct RNA-binding proteins [2,13,14]. In addition, SMN physically contacts translation machinery components and has been proposed as regulator of protein synthesis [15,16,17]. In our previous work, we reported that SMN mediates anchoring of ribosomal proteins (RPs) in caveolin-rich membrane domains and sustains on-site mRNAs translation in an activity-dependent manner [16]. To note, converging evidence highlights a role of SMN in ribosome biogenesis. SMN was found to interact with RP-coding transcripts [18,19,20], whose translation rate appears dysregulated in a mouse model of SMN deficiency [21]. Accordingly, we showed that a reduced level of SMN protein affects both the subcellular distribution and translation efficiency of RPS6 mRNA [20]. Based on these findings, we assumed that SMN might impact the translation pathway early, by selective composition/localization of ribosomes. Moreover, evidence suggests that SMN could contribute to ribosome biogenesis by its direct interaction with nucleolar proteins.

The nucleolus is a membrane-less subcellular compartment in which the ribosome biogenesis takes place [22,23]. It is the nuclear site of ribosomal RNA (rRNA) transcription, pre-rRNA processing, and pre-ribosome subunits assembly [24,25]. Nucleoli contain small nucleolar RNA-protein complexes (snoRNPs). These ancient molecular machines mediate modification and processing of rRNA [26]. To note, it has been shown that SMN binds both fibrillarin and GAR1, two protein components of snoRNPs [25,26]. SMN colocalizes with fibrillarin within dense fibrillary components of nucleoli in HeLa cells, and the presence of SMN in nucleoli was observed in primary tissues as well as in cultured cells [25,27,28,29]. Furthermore, SMN has been found to interact with nucleolin, the major nucleolar protein [30]. Although poorly understood, this intriguing feature of SMN function might be strategic for translational control. Remarkably, nucleolin complexes appear perturbed in fibroblasts from an SMA type I patient [30].

Nucleolin is a highly conserved multifunctional protein implicated in critical cell behaviours, such as proliferation, differentiation, and apoptosis [31,32]. Nucleolin is one of the most abundant proteins in the nucleolus, where it drives pre-rRNA transcription and ribosome assembly [33,34]. Coherently with its multitasking role, nucleolin also localizes in other subcellular regions, including the plasma membrane [35,36]. Nucleolin has a predicted molecular mass of approximately 77 kDa, while its apparent molecular mass of about 100 kDa has been attributed to multiple phosphorylation in the N-terminal domain [37]. Nucleolin is also known as an RNA-binding protein able to affect the fate of target mRNAs in multiple ways [38,39,40,41]. In this regard, an elegant work showed that nucleolin binds and transports the transcript of mammalian target of rapamycin (mTOR) in injured axons [42]. Nucleolin-mediated localization of mTOR mRNA allows local production of mTOR, which is preparatory for a rapid activation of on-site protein synthesis [42]. Therefore, similarly to SMN, nucleolin appears to control translation pathway at different stages, ranging from the production of ribosomal machine to the regulation of translation-related factors, such as mTOR.

To gain further insights into the role of SMN in protein synthesis-related networks, this study explored the SMN-nucleolin interaction. We focused on subcellular distribution of nucleolin as well as its targeted mTOR mRNA. 

We confirmed that SMN physically contacts nucleolin and highlighted, for the first time, an association of SMN with mTOR mRNA. We found that SMN deficiency impairs nucleolar compartmentalization of nucleolin and this correlates with a nuclear retention of mTOR mRNA. We provided evidence that SMN could be required for proper intracellular dynamic of nucleolin–mTOR mRNA complexes. Together, these findings support the notion that SMN may regulate not only ribosomal components of translation machinery, but also their upstream regulating factors.

## 2. Materials and Methods

### 2.1. Antibodies and Reagents

The following antibodies were used: anti-SMN mouse monoclonal antibody (cat. no. 610647, BD Transduction Laboratories, Franklin Lakes, NJ, USA; work dilution for Western blotting, 1:10,000); anti-SMN rabbit polyclonal antibody (cat. no. sc-15320, Santa Cruz Biotechnology, Santa Cruz, CA, USA; work dilution for immunofluorescence, 1:200); anti-C23/nucleolin (MS-3) mouse monoclonal antibody (cat. no.sc-8031, Santa Cruz Biotechnology; work dilution for Western blotting, 1:1000; for immunofluorescence, 1:200); anti-mTOR rabbit polyclonal antibody (cat. no. 2972S, Cell Signaling Technology, Danvers, MA, USA; work dilution for Western blotting 1:500; for immunofluorescence 1:150); anti-Che-1 rabbit polyclonal antibody (work dilution for immunofluorescence 1:200 [43]); anti-UBF1 monoclonal antibody (cat. no.sc-13125, Santa Cruz Biotechnology; work dilution for immunofluorescence, 1:50); anti-alpha-tubulin mouse monoclonal antibody (cat. no. T6074, Sigma-Aldrich, St. Louis, MO, USA; work dilution for Western blotting, 1:2000); anti-puromycin mouse monoclonal antibody (cat. no. MABE343, Millipore, Burlington, MA, USA; work dilution for Western blotting, 1:25,000; for immunofluorescence, 1:1000). The secondary antibodies conjugated to horseradish peroxidase were purchased from Jackson ImmunoResearch Laboratories, West Grove, PA, USA, and used at a dilution of 1:10,000. The Alexa Fluor488- and the Alexa Fluor594-conjugated secondary antibodies were purchased from Thermo Fisher Scientific Inc., Waltham, MA, USA, and were used at a dilution of 1:250.

### 2.2. Cell Cultures and Transfection

hTert-immortalized human fibroblasts (hTert-Fibroblasts) [44] and HeLa human cervical cancer cells were grown in Dulbecco’s modified Eagle’s medium (DMEM, Gibco, Grand Island, NY, USA), supplemented with heat-inactivated 10% FBS (Gibco), penicillin-streptomycin (Gibco) and GlutaMAX (Gibco). SH-SY5Y neuroblastoma cells were grown in DMEM supplemented with heat-inactivated 15% FBS (Australian, Gibco), penicillin-streptomycin (Gibco) and GlutaMAX (Gibco). All cell cultures were maintained at 37 °C in a humidified atmosphere of 5% CO_2_. Transient transfection experiments were performed using Lipofectamine 2000 (Thermo Fisher Scientific Inc.) and a combination of three siRNA-27 duplexes targeting the human SMN1 gene (OriGene, Rockville, MD, USA), following the manufacturer’s instructions. Universal scrambled siRNA duplex was used as negative control. Cells were harvested after 48 or 72 h post transfection.

### 2.3. Co-Immunoprecipitation

Cellular extracts were prepared in IP Buffer (50 mM Tris-HCl pH 7.5, 250 mM NaCl, 5 mM EDTA, 50 mM NaF, 0.1 mM NaVO_4_, 0.1% Triton X-100, 5% glycerol and complete protease and phosphatase inhibitor cocktail (cOmplete, EDTA-free Protease and PhosSTOP tablets, Roche, Indianapolis, IN, USA), and RNase inhibitors (Thermo Fisher Scientific Inc.). Immunoprecipitation assays were performed overnight at 4 °C following standard procedure, using the following antibodies: anti-SMN mouse monoclonal antibody and anti-nucleolin mouse monoclonal antibody. As negative control, the immunoprecipitation was carried out with mouse IgG-beads (Thermo Fisher Scientific Inc.). After five washes in IP buffer the immunoprecipitated complexes were eluted by boiling in Laemmly’s buffer for 10 min and analysed by SDS-PAGE on 10% polyacrylamide gel followed by immunoblotting.

### 2.4. RNA Immunoprecipitation (RIP) Assay

Cells were resuspended in IP Buffer (50 mM Tris-HCl pH 7.5, 250 mM NaCl, 5 mM EDTA, 50 mM NaF, 0.1 mM NaVO_4_, 0.1% Triton X-100, 5% glycerol and complete protease inhibitor cocktail from Roche), in the presence of RNase inhibitors (Thermo Fisher Scientific Inc.). Extracts were vortexed 3 times for 10 s, incubated in ice for 20 min and centrifuged at 10,000× *g* for 7 min at 4 °C. For the immunoprecipitation assay, the protein lysate was pre-cleared with Protein A/G-Agarose beads (Thermo Fisher Scientific Inc.), pre-saturated in 2% BSA-PBS, by replacing beads 3 times within 90 min, at 4 °C. Then 1200 µg of extract was immunoprecipitated in IP buffer overnight with the anti-SMN monoclonal antibody or with anti-nucleolin monoclonal antibody. As negative control, the immunoprecipitation was carried out with mouse IgG-beads (Thermo Fisher Scientific Inc.). The beads were washed five times for 5 min at 4 °C in IP buffer and once in PBS buffer. The immunoprecipitated samples were resuspended in IP buffer. A portion of immunoprecipitation was analysed by Western blot analysis. RNA was extracted using Qiazol reagent (Thermo Fisher Scientific Inc.), according to the manufacturer’s instructions. RNAs were converted to cDNAs using a High-Capacity cDNA Reverse Transcription kit (Thermo Fisher Scientific Inc.).

### 2.5. Cellular Fractionation

Cytoplasmic, nuclear and nucleoli fractions were performed as previously described [45] with little modification. Next, 2 × 100 mm dishes of 80% confluent hTERT-fibroblasts were scraped off while on ice, and collected by centrifugation at 500× *g* for 5 min at 4 °C. After a wash in PBS, cells were resuspended in 1 mL of buffer A (10 mM Hepes pH 7.9, 1.5 mM MgCl_2_, 10 mM KCl, 0.5 mM DTT, and complete protease and phosphatase inhibitor cocktail (cOmplete, EDTA-free Protease and PhosSTOP tablets, Roche) and incubated on ice for 5 min. Extracts were passed 5 times through a 25G needle and centrifugated at 1200× *g* for 5 min at 4 °C. The supernatant was collected and retained as cytoplasmic fraction. The nuclear pellet was resuspended in 0.5 mL of buffer S1 (0.25 M sucrose, 10 mM MgCl_2_) and layered over 0.5 mL of buffer S2 (0.35 M sucrose, 0.5 mM MgCl_2_) and centrifugated at 1200× *g* for 5 min at 4 °C. Clean pelleted nuclei were retained as nuclear fraction or resuspended in 0.5 mL of buffer S2 and sonicated on ice for 6 burst of 10 s, for further subcellular fractionation. The sonicated sample was layered over 0.5 mL of buffer S3 (0.88 M sucrose, 0.5 mM MgCl_2_) and centrifugated at 2800× *g* for 10 min at 4 °C. Nucleoli contained in the pellet were then washed by resuspension in 0.5 mL of buffer S2 followed by centrifugation at 2800× *g* for 5 min at 4 °C. The subcellular fractions were aliquoted and processed for both RNA and protein extractions or for RIP assay.

### 2.6. Western Blot Analysis

Protein extracts were loaded on pre-cast NuPAGE 4–12% gels (Thermo Fisher Scientific Inc.) and transferred onto nitrocellulose membranes (GE Healthcare; Milano, Italy). Immunodetection of the reactive bands was revealed by chemiluminescence (ECL kit, GE Healthcare), and analysed by iBright 1500 (Thermo Fisher Scientific Inc.).

### 2.7. Immunofluorescence Analysis

Immunofluorescence analysis was performed as described previously [16]. Briefly, cells were fixed with 4% formaldehyde in PBS, permeabilized in 0.2% Nonidet P40 (Boehringer Mannheim, Mannheim, Baden-Wurttemberg, Germany) for 20 min and blocked with 1% BSA in PBS at room temperature. Samples were incubated with the primary antibodies, washed three times in PBS and then incubated with the appropriate secondary antibodies. Slides were mounted with ProLong with Dapi (Thermo Fisher Scientific Inc.) and examined by a conventional epifluorescence microscope (Olympus BX53; Milano, Italy). Images were captured by a SPOT RT3 camera and elaborated by IAS 2000 v.5.0.1 software (Biosistem ’82, Rome, Italy). When combined with the padlock assay, the padlock step precedes immunofluorescence procedure.

### 2.8. Padlock Assay

Phosphorylation of the padlock probe and PlaLock assay were performed as previously described [20].

### 2.9. In Situ Proximity Ligation Assay (PLA)

Cells were fixed in 4% PFA for 20 min at room temperature and then permeabilized in 0.2% Nonidet P40 for 20 min. Samples were subjected to in situ PLA using Duolink In Situ Detection Reagents Green kit (DUO92008, Sigma-Aldrich), according to the manufacturer’s instructions. A combination of primary antibodies to SMN (rabbit polyclonal antibody) and nucleolin (mouse monoclonal antibody) were used. PLA signal was detected by epifluorescence microscope (Olympus BX53; Milano, Italy). High-resolution (100× objective) images were analysed by ImageJ (NIH) (v1.8.0, Stapleton, NY, USA) to calculate the density of PLA puncta.

### 2.10. Puro-PLA Assay

Cells were incubated with 10 µM puromycin in the presence of 25 µg/mL Emetine (Sigma-Aldrich) for 10 min. After a wash in PBS, cells were incubated in the fixative/extraction buffer (4% formaldehyde, 0.015% digitonin, 1× PBS) for 20 min at room temperature and then permeabilized in 0.2% Nonidet P40 for 20 min. Therefore, samples were subjected to in situ PLA using Duolink In Situ Detection Reagent Green Kit (DUO92008 Sigma-Aldrich), according to the manufacturer’s instructions. A combination of primary antibodies to puromycin (mouse monoclonal antibody) and mTOR (rabbit polyclonal antibody) was used. PLA signal was detected by epifluorescence microscope (Olympus BX53; Milano, Italy). High-resolution (100× objective) images were analysed by ImageJ (NIH) to calculate the density of PLA puncta.

### 2.11. PlaLock Assay

We generated the “PlaLock” assay combining in situ PLA and padlock methods. Primary antibodies to SMN (rabbit polyclonal antibody) and nucleolin (mouse monoclonal antibody) were incubated with PLA probe anti-rabbit PLUS and PLA probe anti-mouse MINUS, respectively, for 2 h at 37 °C to allow the pre-formation of Abs-plus/minus complexes. Fixed and permeabilized cells were incubated overnight at 37 °C with both the Abs-plus/minus complexes and the specific padlock probe to the mTOR mRNA. The reaction was conducted in 20 µL mixture containing 10 µL of Abs-plus/minus complexes, 2 µL of phosphorylated padlock probe (10 µM), 1 µL of DTT (100 mM), 0.5 µL of RiboLock RNase Inhibitor (Thermo Fisher Scientific Inc.) (40 U µL-1), and 6.5 µL of DEPC-treated H_2_O. The sample was washed twice in PBS/0.01% Tween-20. Then, the reaction of circularization was carried out for 2 h at 37 °C. Cells were incubated with a mixture containing 10 µL of padlock ligase reaction (1X SplintR ligase buffer, 2.5 U µL-1 Splint R ligase, and 1 U µL-1 RiboLock RNase Inhibitor) and 10 µL of PLA ligase reaction (1× ligase buffer, 1.0 U µL-1 ligase). After a wash in PBS/0.01% Tween-20, the RCA primer mixture (0.2 µM RCA primer, 1× SSC, 10% formamide, 5 mM DTT and 0.5 U µL-1 RiboLock RNase Inhibitor) was added to the sample and incubated for 1 h at 37 °C. The RCA reaction was then carried out for 2 h at 37 °C, the sample was incubated with 20 µL of a solution containing 10 µL of padlock RCA reaction solution (1 µL of 10× phi29 DNA polymerase reaction Buffer, 3 µL of dNTPs (10 mM of each dATP, dCTP, dGTP and dTTP), 0.5 µL of phi29 DNA polymerase (10 U µL-1), 0.25 µL of RiboLock RNase Inhibitor (40 U µL-1), and 5.25 µL of DEPC-treated H2O) mixed with 10 µL of PLA amplification reaction solution (2 µL of 5X Amplification stock, 0.125 µL of polymerase (10 U µL-1) and 7.875 µL of DEPC-treated H_2_O). The incubation was followed by two washes of 5 min each in PBS/0.01% Tween-20. Finally, the sample was incubated with 100 nM fluorophore-labelled detection probe in 2× SSC and 15% formamide for 30 min at 37 °C. After three washes in PBS/0.01% Tween-20 for 5 min each, slides were mounted as reported above.

### 2.12. Quantification and Statistical Analysis

All experiments were performed on at least three independent biological replicates. Data are presented as mean ± s.d. Statistical analysis was performed using the GraphPad Prism software. Data were analysed using an unpaired *t*-test or a two-way ANOVA test with Bonferroni test for multiple comparison as specified in the figure legends; *p* < 0.01 was considered statistically significant.

### 2.13. Oligos

Oligos used in this study are indicated in Table S1.

## 3. Results

### 3.1. SMN Interacts with Nucleolin

**Figure 1:**
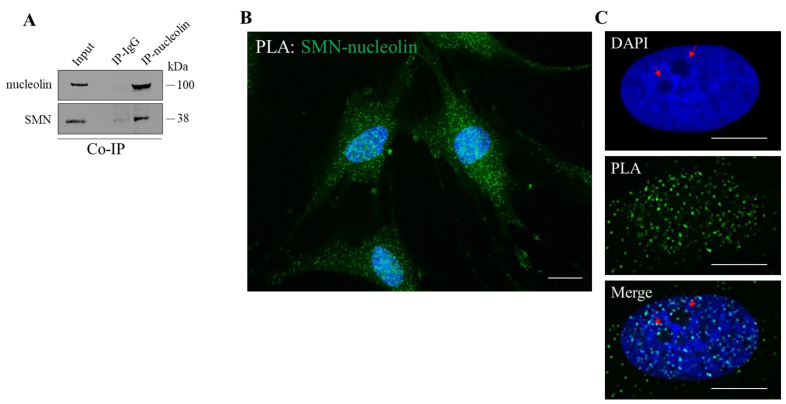
SMN interacts with nucleolin. (**A**) Cellular extracts from hTert-Fibroblasts were processed for co-immunoprecipitation assay (Co-IP) using nucleolin monoclonal antibody-conjugated beads (nucleolin Ab) or mouse IgG-conjugated beads (IgG), as negative control. Then, samples were subjected to Western Blot analysis. The 5% of the protein extract was used as input. Representative immunoblotting, of *n* = 3 independent experiments showing the co-precipitation of SMN with nucleolin. (**B**) Representative image of in situ proximity ligation assay (PLA) performed in hTert-Fibroblasts using primary antibodies against SMN and nucleolin (rabbit polyclonal Ab and mouse monoclonal Ab, respectively). PLA puncta (green dots) are indicative of SMN-nucleolin interaction sites. Nuclei were stained with DAPI (blue). Scale bar 10 µm. (**C**). In detail, a representative nucleus labelled with DAPI. Nucleoli are typically visible as dark regions due to low DNA density (red arrows). As showed in the Merge panel, some PLA dots are visible around and within the nucleoli. Scale bars, 5 µm.

We aimed to further elucidate the relationship between SMN and nucleolin, due to their strategic role in translational control. Based on previous studies showing the association between SMN and nucleolin [30], we also verified this event in our experimental system. By a co-immunoprecipitation assay we showed that a pool of endogenous SMN protein co-precipitates with nucleolin in cellular extracts from immortalized human fibroblasts (hTert-Fibroblasts) (Figure 1A). Furthermore, we carried out in situ proximity ligation assays (PLA) to visualize where SMN directly interacts with nucleolin (Figure 1B). By fluorescence microscopy, we visualized several SMN–nucleolin interaction dots in nuclei as well as throughout the cytoplasm, including peripheral regions of the labelled fibroblasts. Some PLA dots were also detectable within and/or around the nucleolus (Figure 1C). PLA specificity was validated by performing appropriate controls (Figure S1). Moreover, similar PLA dots distribution was found in human neuroblastoma SH-SY5Y, and HeLa cells (Figure S2).

### 3.2. SMN Mediates Nucleolar Compartmentalization of Nucleolin

**Figure 2:**
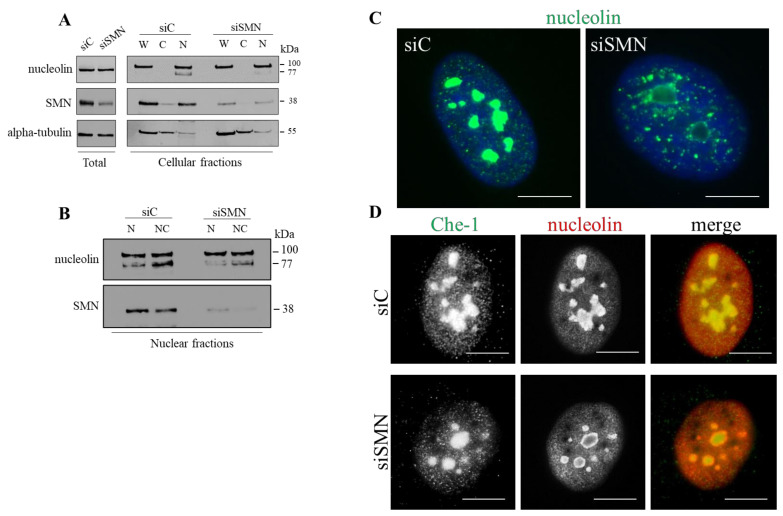
SMN deficiency impairs nucleolar localization of nucleolin. (**A**,**B**) hTert-Fibroblasts, transiently transfected with SMN1 siRNA (siSMN) or scrambled siRNAs, as control (siC), were subjected to subcellular fractionation. (**A**) Immunoblotting shows the abundance of the indicated proteins in each subcellular fraction (W = whole-cell extract; C = cytoplasm; N = nucleus); (**B**) nuclei (N) and nucleoli (NC) isolated from the transfected fibroblasts were checked by immunoblotting. Representative panels of *n* = 3 independent experiments. (**C**,**D**). Representative immunofluorescence of siSMN- or siC-transfected hTert-Fibroblasts; (**C**) Cells were immunostained for nucleolin (green). Scale bar 5 µm. (**D**) The transfected fibroblasts were subjected to dual immunostaining for the nucleolar marker Che-1, and nucleolin (green and red, respectively, in the merge). Scale bar 5 µm. In **C** and **D**, nuclei were stained with DAPI (blue).

The next step was to understand whether SMN might be implicated in subcellular localization of nucleolin. We downregulated expression levels of SMN in hTert-Fibroblasts. Cells were transiently transfected with a pool of SMN1-selective siRNAs (siSMN). Scrambled siRNAs were used as control (siC). Then, 48 h after transfection, total protein extracts were subjected to Western blot analysis to evaluate nucleolin-related products. A 100 kDa band was detected, without significant differences between control and SMN-depleted cells (Figure 2A, total, and Figure S3). Changes upon SMN depletion were finely revealed at the subcellular level. Transfected fibroblasts were processed to obtain cellular subfractions, whose immunoreactivity to anti-nucleolin antibody was checked by Western blot analysis. Nucleolin was present in both the whole and nuclear compartment (Figure 2A, W = whole, N = nucleus). Upon SMN knockdown, the expression levels of the 100 kDa protein appeared unchanged, while the intensity of the 77 kDa band, detectable following nuclei enrichment, appeared decreased (Figure 2A,N). This intriguing result prompted us to further dissect the nuclear fraction. Indeed, nucleolin is mostly abundant in nucleoli that, due to their peculiar chemical features, may be easily isolated from nuclei [45]. Nucleoli isolated from siC- and siSMN-transfected fibroblasts were subjected to Western blot analysis to monitor nucleolin levels (Figure 2B, NC = nucleolus). By this biochemical approach, we assumed that: (1) at least two products of nucleolin, with an apparent molecular weight of 100 and 77 kDa, were identified in nucleoli of human immortalized fibroblasts; (2) the 77 kDa band preferentially enriched in nucleoli, and most importantly, (3) its detection was specifically reduced by SMN knockdown. The subcellular distribution of nucleolin was also explored by immunofluorescence assays carried out in both siC- and siSMN-transfected fibroblasts (Figure 2C,D, Figure S4). To identify SMN-deficient cells an immunostaining for SMN was performed in parallel. As reported in previous works [16,20], no fluorescence signals above background levels were detectable in approximately 80–85% of siSMN-transfected fibroblasts (data not shown). In control cells, nucleolin immunostaining exhibited a peculiar localization pattern, characterized by a strong fluorescent signal from nucleoli. Granular staining from extra-nucleolar regions of nuclei was also observed (Figure 2C, siC). Moreover, consistent with our PLA images—as well as with the notion that nucleolin is a shuttle protein—its cytoplasmic localization was detectable after a long exposure time (Figure S4). By comparative analysis, substantial differences were found in nucleoli of SMN-deficient cells, where nucleolin staining was clearly impaired (Figure 2C, siSMN). Instead, the immunostaining of Che-1, a known nucleolar marker [46,47], appeared almost unchanged (Figure 2D). We also monitored an additional nucleolar protein, the Upstream Binding Transcription Factor (UBF1), whose immunostaining was almost unaffected by SMN-deficiency (Figure S5). Furthermore, at this stage, we could not exclude partial redistribution of nucleolin in subnuclear regions.

Collectively, these results confirm that SMN interacts with nucleolin and suggest that SMN might be required for proper nucleolin compartmentalization.

### 3.3. SMN Coexists with Nucleolin–mTOR mRNA Complexes

**Figure 3:**
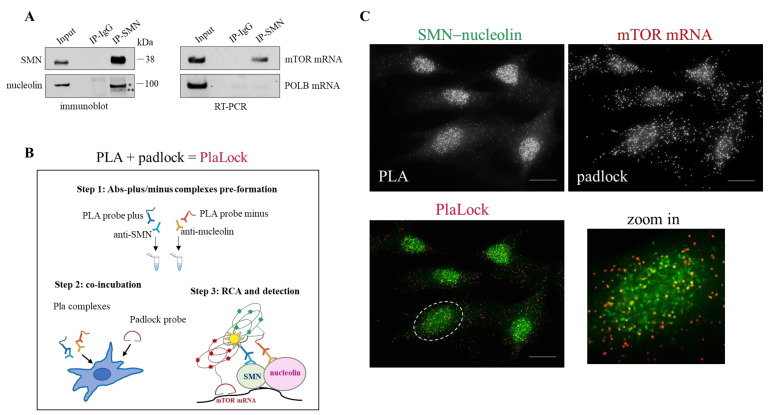
SMN coexists with nucleolin–mTOR mRNA complexes. (**A**) Cellular extracts from hTert-Fibroblasts were subjected to RNA-immunoprecipitation (RIP) assay. SMN monoclonal antibody-conjugated beads or mouse IgG-conjugated beads were used in immunoprecipitation steps. (left) Immunoblotting validating nucleolin co-precipitation with SMN. To note, anti-nucleolin antibody detects two bands: a main band of 100 kDa (*) and a faint, smaller band (**). (right) mTOR mRNA were checked in RIP samples by RT-PCR analysed by agarose gel electrophoresis. DNA Polymerase Beta (POLB) mRNA was monitored as control. Representative panels of *n* = 3 independent experiments. (**B**) Diagram illustrating the main experimental steps of the “PlaLock” assay in fixed cells. PlaLock assay was designed to combine the PLA and padlock methods in situ. In this way, it is possible to visualize the coexistence, at subcellular levels, of a selected mRNA with a specific protein–protein complex (see Methods section). (**C**) Representative images of a PlaLock performed in hTert-Fibroblasts. (right) SMN-nucleolin complexes visualized by PLA. (left) Subcellular distribution of mTOR mRNA by padlock. The bottom panel visualizes the merged images. mTOR mRNA and SMN-nucleolin complexes are labelled in red and green, respectively. Yellow dots are mostly detectable in nuclear regions, as illustrated in the higher magnification of the white dotted circle region (zoom in). Scale bar 5 µm.

Given that nucleolin is an RNA-binding protein, we asked whether SMN can associate with transcripts targeted by nucleolin. Among mRNAs targeted by nucleolin and critically implicated in translation pathway, those encoding for mTOR caught our attention. To verify a potential physical link between SMN and mTOR transcript we approached RNA-immunoprecipitation (RIP) experiments. The fact that nucleolin binds mTOR mRNA was showed so far in a mouse model of sciatic nerve injury. By RIP, using an anti-nucleolin monoclonal antibody, we confirmed that mTOR mRNA is also physically targeted by nucleolin in human fibroblasts (Figure S6). Then, by using an anti-SMN antibody, we showed that mTOR mRNA co-precipitates with SMN, together with nucleolin (Figure 3A). Coherently, immunofluorescence microscopy revealed partial overlapping of SMN immunostaining and rolling circle amplification (RCA) dots generated by a padlock assay targeting mTOR transcripts (Figure S7). As previously reported, padlock probes, coupled to RCA, allow a specific transcript to be labelled with a near-single-molecule resolution [48].

Next, our intent was to visualize subcellular sites in which SMN contacts nucleolin–mTOR mRNA complexes. To do this, we developed a new assay that we named “PlaLock”. This assay combines in situ PLA and padlock methodologies in fixed cells (Figure 3B). In this way, it is possible to provide colocalization images of a single mRNA with a selected protein–protein complex. We designed a padlock probe—which specifically target mTOR mRNA—and used antibodies against SMN and nucleolin in the PLA step. As schematically illustrated in Figure 3B, to optimize our strategy we partially modified the canonical PLA procedure. Fixed fibroblasts, subjected to PlaLock assay, were then analysed by high-resolution fluorescence microscopy (Figure 3C). A combination of different controls was performed to confirm the specificity of PlaLock (Figure S8). By monitoring RCA dots, we found that mTOR mRNA was diffusely distributed throughout the cytoplasm and nuclear/perinuclear regions of the cell (Figure 3C, *padlock*). PLA images confirmed the subcellular distribution/frequency of SMN–nucleolin complexes (Figure 3C, *PLA*). In line with RIP results, mTOR mRNA partially overlapped with PLA dots, as clearly indicated by yellow puncta (Figure 3C, *PlaLock*). These overlapped dots were mainly detectable in nuclei of the labelled fibroblasts.

These findings highlight for the first time an interaction between SMN and mTOR transcript. By using an innovative PlaLock assay, we provide evidence that a pool of the SMN protein traffics in association with nucleolin–mTOR mRNA complexes.

### 3.4. SMN Deficiency Delocalizes mTOR mRNA and Perturbs its Translation

**Figure 4:**
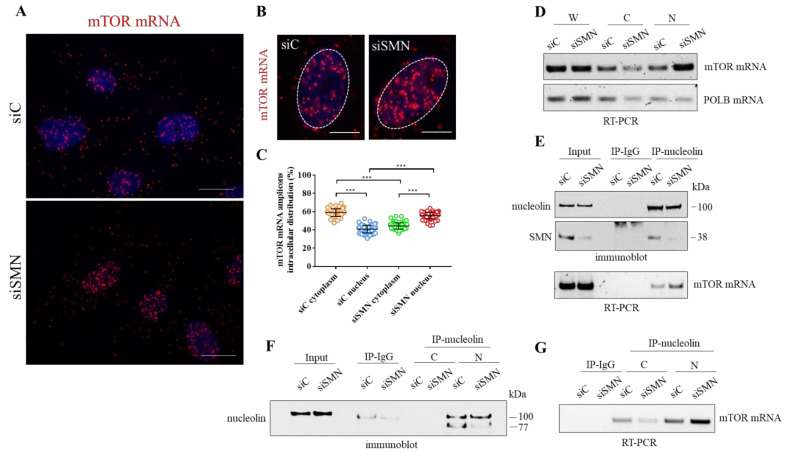
Subcellular distribution of mTOR mRNA. (**A**) Representative images of padlock assay targeting mTOR mRNA (red dots), in siC- or siSMN-transfected hTert-Fibroblasts. Nuclei were stained with DAPI. Scale bar 10 µm. (**B**). Representative images visualizing the nuclear localization of mTOR mRNA (red dots) in siSMN-transfected cells, in comparison with the control (siC). Nuclei were labelled with DAPI. Scale bar 5 µm. (**C**) Scatterplot showing the percentage of mTOR mRNA amplicons distributed in the cytoplasm or nuclei of the transfected fibroblasts (*n* = 20 cells were analysed for each condition. In graph are plotted all the results from four independent experiments, *** *p* < 0.0001 two-way ANOVA–Bonferroni’s multiple comparisons test). Mean ± s.d. are illustrated. (**D**) Subcellular fractionation of hTert-Fibroblasts. The distribution of mTOR mRNA and DNA Polymerase Beta (POLB) mRNA in different subcellular fractions (W = whole cell extract; C = cytoplasm; N = nucleus) were checked by RT-PCR and analysed by agarose gel electrophoresis. Representative gel of *n* = 3 independent experiments. (**E**) Cellular extracts from both the transfected fibroblasts were subjected to RIP assay. Nucleolin monoclonal antibody-conjugated beads or mouse IgG-conjugated beads were used in the immunoprecipitation step. Co-precipitation of nucleolin and SMN are displayed by immunoblotting. The presence of mTOR mRNA in RIP samples was checked by RT-PCR analysed by agarose gel electrophoresis. Representative panels of *n* = 3 independent experiments. (**F**,**G**) Cytoplasmic (C) or nuclear (N) fractions from the transfected fibroblasts were subjected to RIP assay. Nucleolin monoclonal antibody-conjugated beads or mouse IgG-conjugated beads, were used in the immunoprecipitation step. (**F**) Immunoblotting validating the efficiency of nucleolin immunoprecipitation. (**G**) The presence of mTOR mRNA in each fraction was checked by RT-PCR analysed by agarose gel electrophoresis. Representative panels of *n* = 3 independent experiments.

**Figure 5:**
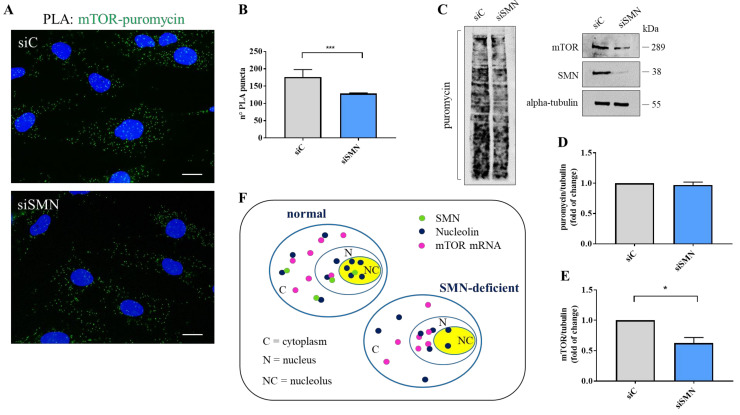
SMN knockdown reduces neo-synthesis of mTOR. (**A**) Puro-PLA assay was carried out using primary antibodies against puromycin and mTOR (mouse monoclonal and rabbit polyclonal, respectively). Representative images visualizing the subcellular distribution of newly synthesized mTOR protein (green dots) in both siC- and siSMN-transfected fibroblasts. Nuclei were stained with DAPI (blue). Scale bars, 10 µm. (**B**) Quantitative analysis of Puro-PLA puncta per cell (*n* = 20 cells were analysed for each sample. *** *p* < 0.0001 one-way ANOVA–Bonferroni’s multiple comparisons test (Mean ± s.d.; *n* = 3) (**C**) Evaluation of protein synthesis rate by SUnSET assay of both siC- and siSMN-transfected fibroblasts. Equal amounts of proteins were immunoblotted with the indicated antibodies. (**D**) Densiometric analysis of puromycin normalized to tubulin. No statistically significant changes are observed in hTERT-fibroblast transfected with siSMN1 siRNA (siSMN) compared with hTERT-fibroblast transfected with the control (siC). (Mean ± s.d.; *n* = 3; Student’s *t*-test.) (**E**). Densiometric analysis of mTOR normalized to tubulin. Statistically significant changes occurring in SMN-depleted cells (siSMN), compared to the control (siC). (Mean ± s.d.; *n* = 3; * *p* = 0.01, Student’s *t*-test). (**F**) Graphical representation for the mechanism by which SMN fine-tunes nucleolin and mTOR mRNA complexes.

To gain further insights into the relationship between SMN and mTOR mRNA, we carried out padlock experiments in both siC- and siSMN-transfected fibroblasts. Then, we compared subcellular localizations of mTOR mRNA by fluorescence microscopy (Figure 4A,B). In control cells (siC), mTOR mRNA appeared diffusely distributed throughout the cytoplasm as well as in the nuclear/perinuclear regions. Remarkably, mTOR mRNA clearly enriched in nuclei of SMN-deficient cells (Figure 4B). By quantitative analysis of amplicons distribution, we evaluated that, contextually to nuclear enrichment, mTOR mRNA appeared reduced in cytoplasm of SMN knockdown cells (Figure 4C). Thus, we assumed that SMN deficiency caused redistribution, rather than a global increase, of mTOR mRNA. Indeed, it has already been shown that SMN deficiency does not change expression levels of mTOR transcript [49]. To further assess a potential delocalization of mTOR transcript, we took advantage of biochemical fractionation of the transfected fibroblasts. We purified the RNA content of the whole, cytoplasm, and nuclei of both siC- and siSMN-transfected cells. Then, using RT-PCR analysis, we checked and compared the abundance of mTOR mRNA in each fraction (Figure 4D, Figure S9). In the whole fraction, SMN silencing resulted in non-significant changes about mTOR mRNA levels (Figure 4D). Conversely, in comparison with the control, siSMN cells exhibited enrichment of mTOR transcript in nuclei, arguing a shift of this transcript from the cytoplasm to nuclear regions. Overall, this biochemical approach agreed with our imaging data, confirming subcellular redistribution of mTOR transcript occurring in SMN-deficient cells. Next, we asked whether SMN knockdown could affect the association between nucleolin and mTOR mRNA. Total cellular extracts of both siC- and siSMN-transfected fibroblasts were subjected to RIP assay by using anti-nucleolin monoclonal antibody (Figure 4E). As shown in Figure 4 panel E, SMN deficiency increased co-precipitation of mTOR mRNA with nucleolin, and this event could not be explained by an improvement of nucleolin pull-down (Figure 4E, IP-nucleolin). Since our findings highlighted that SMN function impacts mainly at a subcellular level [16,20], we repeated RIP experiments following subcellular fractionation (Figure 4F). Remarkably, the amount of mTOR mRNA that co-immunoprecipitated with nucleolin was higher in nuclei isolated from siSMN-transfected fibroblasts compared with the control. Then, we suspected that SMN could affect not only the subcellular sorting of mTOR transcript, but also its translation rate. To verify this hypothesis, we carried out a Puro-PLA assay [50]. This method couples puromycylation with a proximity ligation assay allowing visualization of a newly synthetized protein in single cells. The distribution of PLA puncta that identified newly synthetized mTOR was evaluated by fluorescence microscopy (Figure 5A, Figure S10). In control cells (Figure 5A, siC), we detected several PLA puncta throughout the cytoplasm, including peripheral regions, and nuclear/perinuclear sites. A similar pattern was also found in siSMN-transfected fibroblasts (Figure 5A, siSMN), although the frequency of PLA puncta appeared lower in comparison to the control. Using quantitative analysis, we confirmed that SMN knockdown significantly decreases intracellular hotspots of mTOR neo-synthesis (Figure 5B). Translation rate of mTOR was also evaluated by SUnSET assay [51] (Figure 5C). Puromycin incorporation in both siC- and siSMN-transfected fibroblasts was quantified by densitometric analysis of the corresponding immunoblot (Figure 5D). No significant difference was revealed, confirming that SMN deficiency does not affect global protein synthesis in steady-state conditions. Despite of this, SMN depletion caused a significant reduction in mTOR protein (Figure 5E).

These findings strongly suggest that SMN-deficient cells might prevent aberrant translation events by reducing cytoplasmic availability of mTOR transcript. Interestingly, in this context nucleolin might undergo to a switch of its function/localization, contributing to the recruitment of mTOR mRNA in nuclei (Figure 5F).

## 4. Discussion

Accurate tuning of gene expression levels relies on sophisticated mechanisms that orchestrate RNA localization and translation in space and time [52]. Neo-synthesis of translation machinery components appears itself spatially regulated. We previously suggested that SMN might contribute to ribosome biogenesis by promoting subcellular localization and translation of RP-coding transcripts [20]. In human fibroblasts, SMN traffics with a pool of RPS6 mRNA, targeting its subcellular localization and translation [20]. In line with this notion, Bernabò and colleagues showed a perturbed translation efficiency of RP-coding transcripts in a mouse model of SMA [21]. Therefore, SMN appears involved at different steps of the translational pathway, ranging from ribosome biogenesis to local translation. In this framework, a direct interaction of SMN with non-ribosomal factors of the translational control could represent an additional level of regulative complexity.

Experimental evidence highlighted a functional link between nucleolar processes and SMN. A dynamic association has been demonstrated between SMN with fibrillarin and GAR1 [25,26]. Both these proteins are specific components of snoRNPs, which are conserved throughout evolution and mediate transcription/maturation of rRNAs [24]. Remarkably, snoRNPs are depleted from the nucleolus in cells expressing a dominant–negative mutant of SMN [26], and this implies a role of SMN not only in the biogenesis of snoRNPs, but also in nucleolar architecture/composition. Nucleolin, one of the most abundant non-ribosomal proteins in the nucleolus, critically drives ribosome biogenesis [31,37]. It has been reported that SMN interacts with nucleolin [30]. Given the crucial role of these RNA-related proteins in translational control, here we verified a potential relationship between SMN and nucleolin. We observed a direct SMN–nucleolin interaction in human immortalized fibroblasts, neuroblastoma SH-SY5Y, and HeLa cells. Co-immunoprecipitation experiments, using total extracts from fibroblasts, validated the existence of SMN–nucleolin complexes. As expected, our results confirmed a previous work showing an association of SMN with nucleolin in nuclear extracts from COS-7 cells, SV40-immortalized human fibroblasts, and mouse spinal cord [30]. Our PLA images also suggest that a pool of SMN protein could contact nucleolin into and/or around the nucleolus. Indeed, nucleolar localization of SMN was already observed in postnatal tissues [25,27,28,29]. However, this event appears difficult to observe, probably because SMN associates with nucleoli transiently. Furthermore, fibroblasts derived from a patient affected by a severe form of SMA exhibit disrupted nucleolin complexes [30]. Accordingly, we provide evidence that SMN deficiency could interfere with the canonical localization of nucleolin. In support of this, we took advantage of biochemical isolation of nucleoli. In our system, a band of 77 kDa, corresponding to the predicted molecular weight of nucleolin, was mainly detectable in nucleoli. An additional band of nucleolin, with an apparent molecular weight of 100 kDa, was detectable in both nucleolar and extra-nucleolar regions of the cell. SMN knockdown specifically decreased the detection of 77 kDa nucleolin. Conversely, 100 kDa nucleolin resulted as apparently unaffected by SMN depletion. Concurrently, the immunofluorescence analysis confirmed that SMN deficiency strongly impairs the localization of nucleolin in nucleoli (Figure 5F). Concerning this issue, it is important to mention that phosphorylation may change subcellular distribution of nucleolin. It has been shown that extra-nucleolar traffic of nucleolin requires its phosphorylation via the PI3K/Akt pathway [53]. Thus, it is possible that SMN could interact dynamically with different nucleolin complexes in distinct subcellular compartments, as supported by PLA images. However, SMN deficiency could impact mainly on a pool of newly synthetized nucleolin preventing its nucleolar compartmentalization. We need further studies to test this hypothesis.

As known, nucleolin is an RNA-binding protein that targets several mRNAs [39]. Terenzio et al. showed that mTOR mRNA traffics with nucleolin in injured axons [42]. Locally translated mTOR activates the neo-synthesis of appropriate factors, allowing a rapid shift of the axonal proteome [42]. Therefore, linkage between nucleolin and mTOR, two master regulators, may become strategic in translational control. We suspected that SMN could mediate this sophisticated interplay. It is noteworthy that we showed, for the first time, a physical association between SMN and mTOR transcripts. Next, by an innovative PlaLock assay, we visualized subcellular sites where SMN physically contacts nucleolin–mTOR mRNA complexes. Importantly, we found that a reduced level of SMN protein induces a nuclear recruitment of mTOR mRNA, as well as its reduced translation efficiency. Moreover, we provide evidence that a subset of nucleolin could contribute to retain mTOR mRNA in nuclei upon SMN deficiency. To note, we previously reported that RPS6 mRNA also accumulates in nuclei of SMN-deficient cells [20]. We suppose that in a context of SMN deficiency, characterized by protein synthesis alteration, the nuclear recruitment of translation-related transcripts could be a mechanism to prevent deleterious translation events. Concerning this issue, we previously demonstrated that SMN-deficient cells retain the ability to activate mTOR pathway under specific stimulation [16,20]. However, the stimulated cells displayed a reduction in translation rate upon SMN depletion. We thus assumed that SMN could facilitate a subset of protein synthesis downstream of mTOR activation. Indeed, further studies showed that SMN deficiency affects mainly ribosomal proteins synthesis [20,21]. Notably, all the mRNAs translating for ribosomal proteins are specifically regulated by mTOR [54]. Based on our data, it is reasonable to suppose that SMN might govern not only structural components of the translation machinery, but also its upstream regulating factors.

Collectively, these findings demonstrate that SMN modulates protein composition of the nucleolus by targeting its most abundant component, nucleolin. We provide evidence that SMN fine-tunes the sophisticated interplay between nucleolin and mTOR, two master regulators of translation platforms (Figure 5F). In agreement with our previous work [20], we suggest that SMN-deficient cells might prevent aberrant translation events by reducing cytoplasmic availability of translation-related transcripts. This study highlights the wide complexity of the SMN role in translational control and points to SMN as a key determinant, able to regulate protein synthesis from near and far.

## Data Availability

Data are contained within the article or Supplementary Materials.

## Supplementary Materials

The following are available online at https://www.mdpi.com/article/10.3390/cells10113015/s1, Figure S1: SMN–nucleolin interaction; Figure S2: Densitometric analysis; Figure S3: Nucleolin localization, Figure S4: RIP Assay; Figure S5: Immunofluorescence Analysis of UBF1; Figure S6: RIP Assay; Figure S7: Dual staining for SMN and mTOR mRNA; Figure S8: PlaLock controls; Figure S9: Dual staining for SMN and mTOR mRNA; Figure S10: Puro-PLA control; Table S1: Oligos used in this study.
