# Supplementary material for: Fine-Tuning of mTOR mRNA and Nucleolin Complexes by SMN
Source: Cells. 2021 Nov 4;10(11):3015. doi: 10.3390/cells10113015 (PMC8616268; doi:10.3390/cells10113015)
Supplement: Supplementary file 1 [file cells-10-03015-s001.zip › cells-1395185-supplementary.pdf]

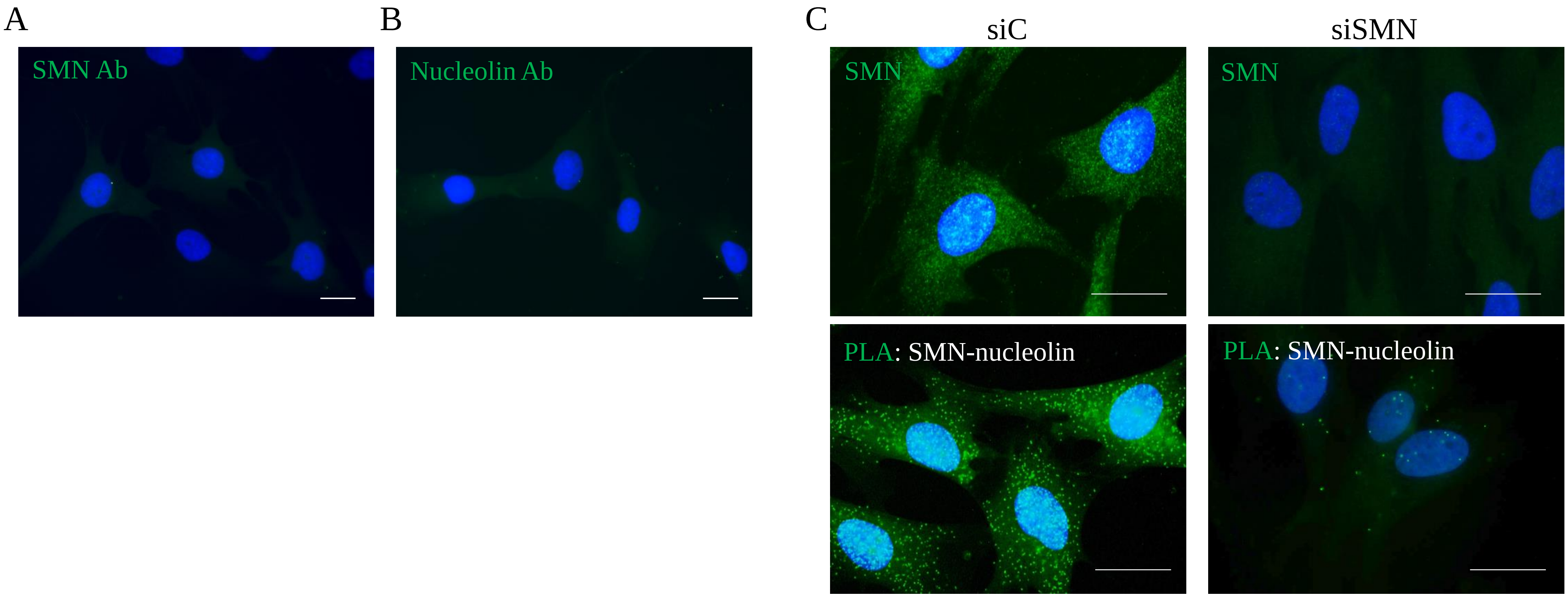

Figure S1. PLA negative controls. Representative images of in situ proximity ligation assay (PLA) performed in hTERT-fibroblasts. PLA specificity was validated by omitting one of the primary antibodies in the assay. (**A**) Only the primary antibody against SMN was used in the assay (SMN Ab) . (**B**) Only the primary antibody against nucleolin was used in the assay (Nucleolin Ab) . Nuclei were stained with DAPI (blue). Scale bar 10 µm. (**C**) SMN-depleted hTert-Fibroblasts. Cells were transiently transfected with a pool of SMN1-selective siRNAs (siSMN). Scrambled siRNAs were used as control (siC). 48 hours after transfection, PLA was carried out by using antibodies against SMN and nucleolin. In parallel, the SMN depletion was verified by performing an immunostaining with a monoclonal antibody against SMN. Scale bar 10 µm.

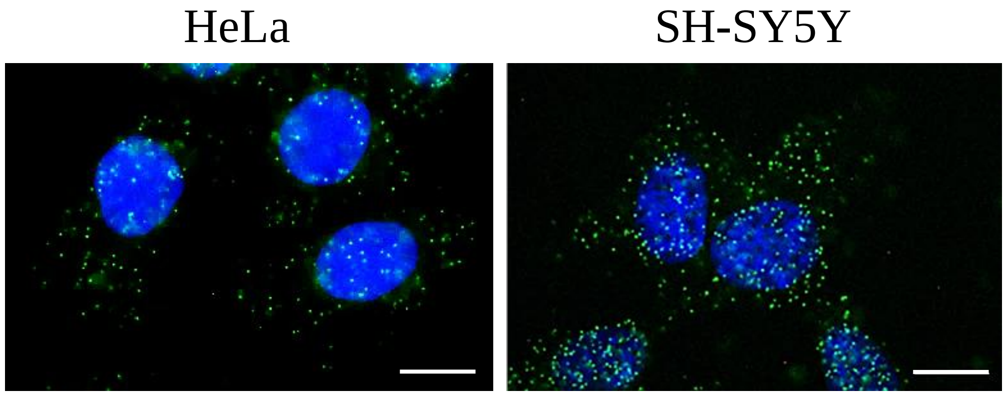

Figure S2. SMN-nucleolin interaction. Representative images of in situ proximity ligation assay (PLA) performed in HeLa and SH-SY5Y cells, using primary antibody against SMN and nucleolin (polyclonal and monoclonal, respectively). PLA dots (green) are indicative of SMN-nucleolin interaction sites. Nuclei were stained with DAPI (blue). Scale bar 10 mm.

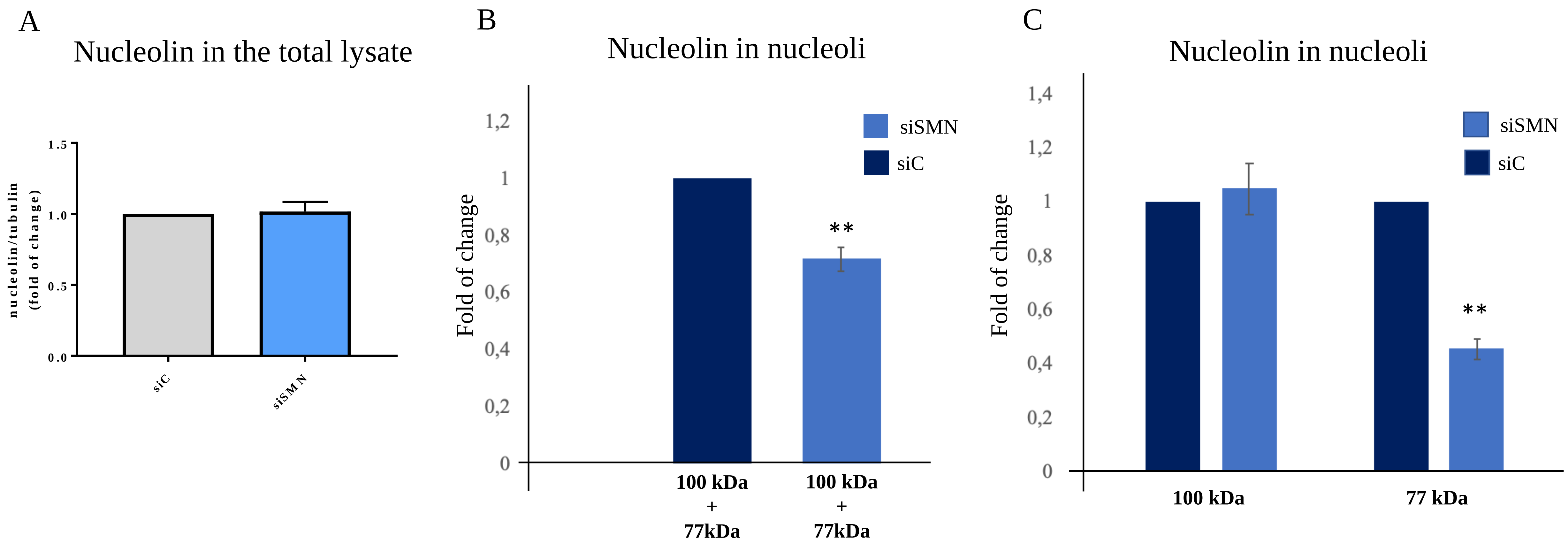

Figure S3. Densitometric analysis. (**A**) Densitometric analysis of nucleolin immunoblot in total lysate normalized to alpha-tubulin. No statistically significant changes are observed in SMN-deficient fibroblasts (siSMN) compared to the control (siC). (Mean ± s.d.; n=3; Student's t test). (**B**) Relative densitometric analysis of nucleolin bands detected in nucleolus from SMN-deficient fibroblasts (siSMN) compared to the control (siC). (Mean ± s.d.; n=3; Student's t test). (**C**) Relative densitometric analysis of 100 kDa nucleolin and 77 kDa nucleolin in nucleoli from SMN-deficient fibroblasts (siSMN) compared to the control (siC). (Mean ± s.d.; n=3; Student's t test).

Figure S4. Nucleolin localization. Immunostaining of nucleolin (green) in siC- or siSMN-transfected fibroblasts. Nuclei were stained with DAPI (blue). Short or long exposure is illustrated. To note, long exposure displays a granular staining throughout the cytoplasm of the labelled fibroblasts. Scale bar 10 µm.

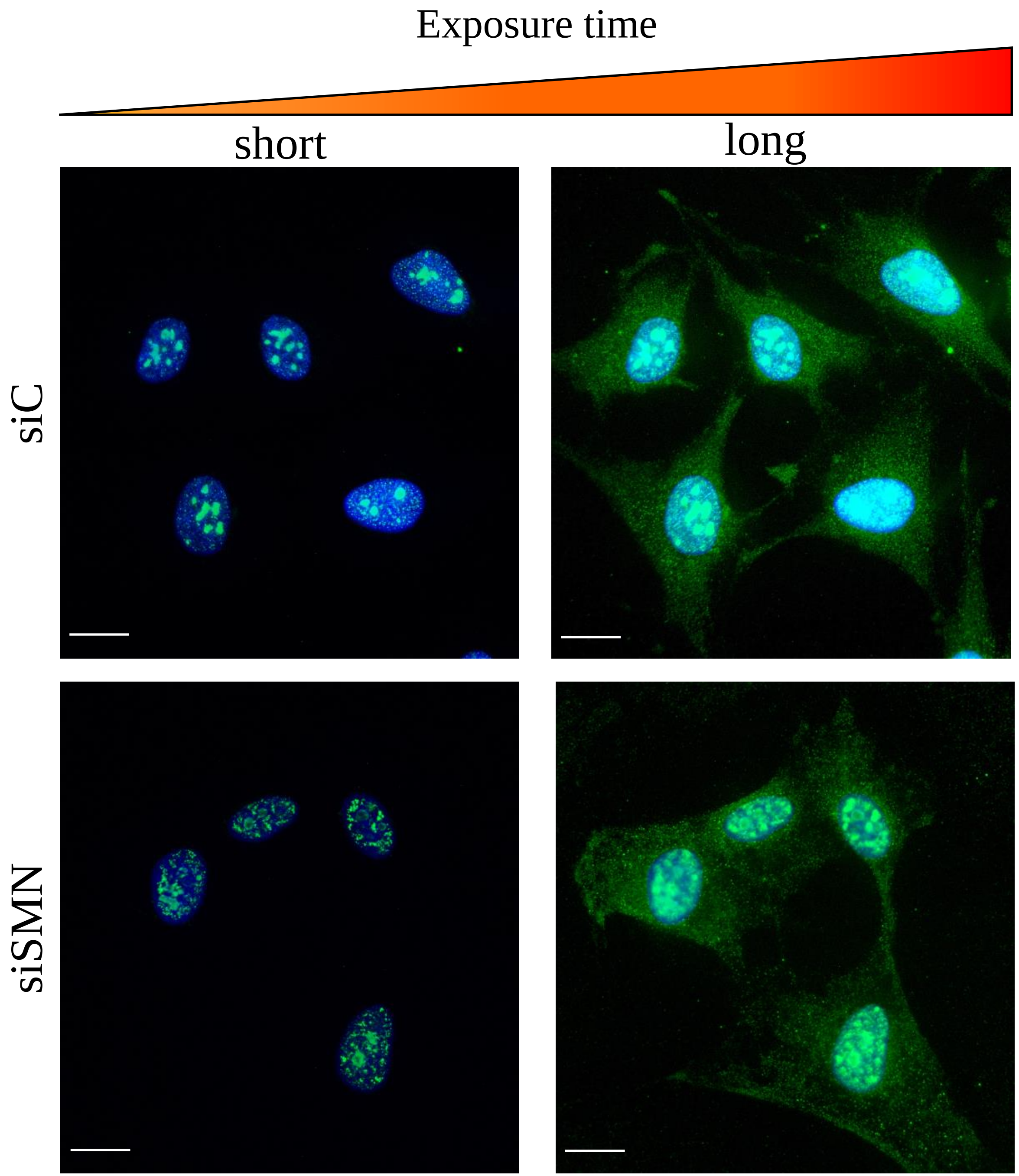

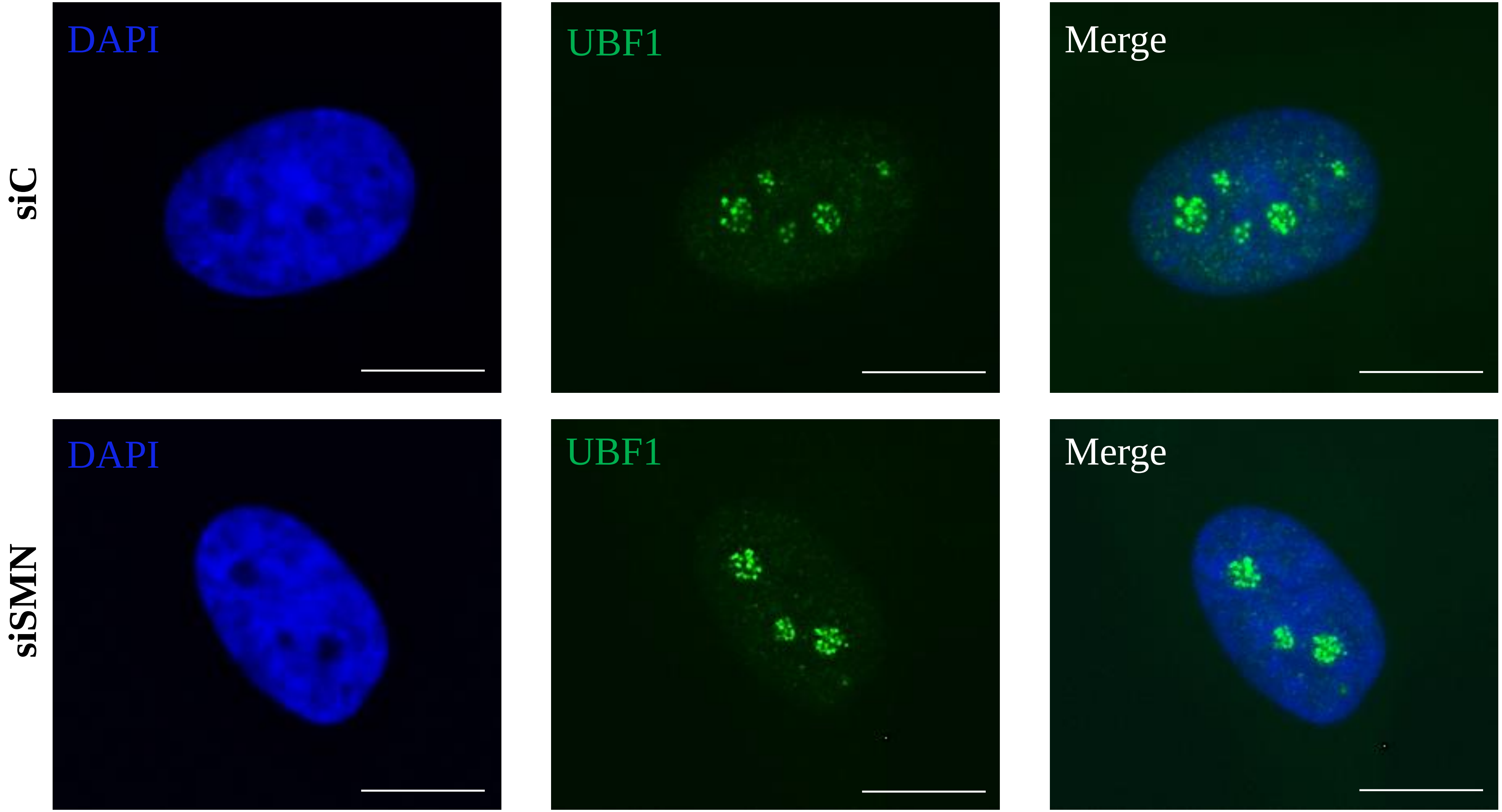

Figure S5. Immunofluorescence Analysis. Representative images of siC-transfected or siSMN-transfected fibroblasts subjected to immunostaining with an anti-UBF1 monoclonal antibody. Nuclei were labelled with DAPI. Scale bar 5 µm.

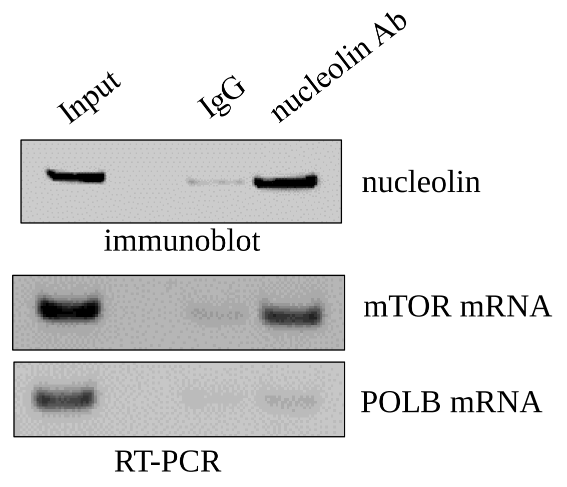

Figure S6. RIP Assay. Cellular extracts from hTERT-fibroblasts were processed for RNA-immunoprecipitation (RIP) assay. Nucleolin monoclonal antibody-conjugated beads or mouse IgG conjugated beads were used in immunoprecipitation step. Immunoblotting validating the efficiency of nucleolin immunoprecipitation. mTOR mRNA and DNA Polymerase Beta (POLB) mRNA in RIP samples were checked by RT-PCR, analysed by agarose gel electrophoresis.

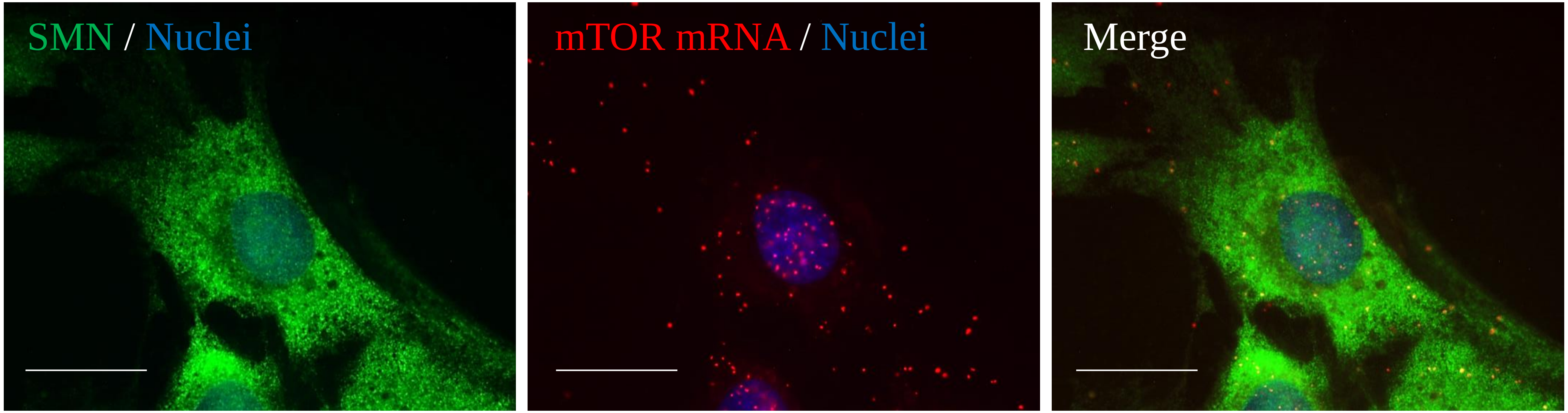

Figure S7. Dual staining for SMN and mTOR mRNA. hTert-Fibroblasts were subjected to a combination of SMN immunostaining (green) and padlock assay targeting mTOR mRNA (red), and then were imaged by high-resolution fluorescence microscopy. Representative images showing overlapped fluorescent signals (yellow dots in the Merge). Nuclei were labelled with DAPI (blue). Scale bar, 10 μm.

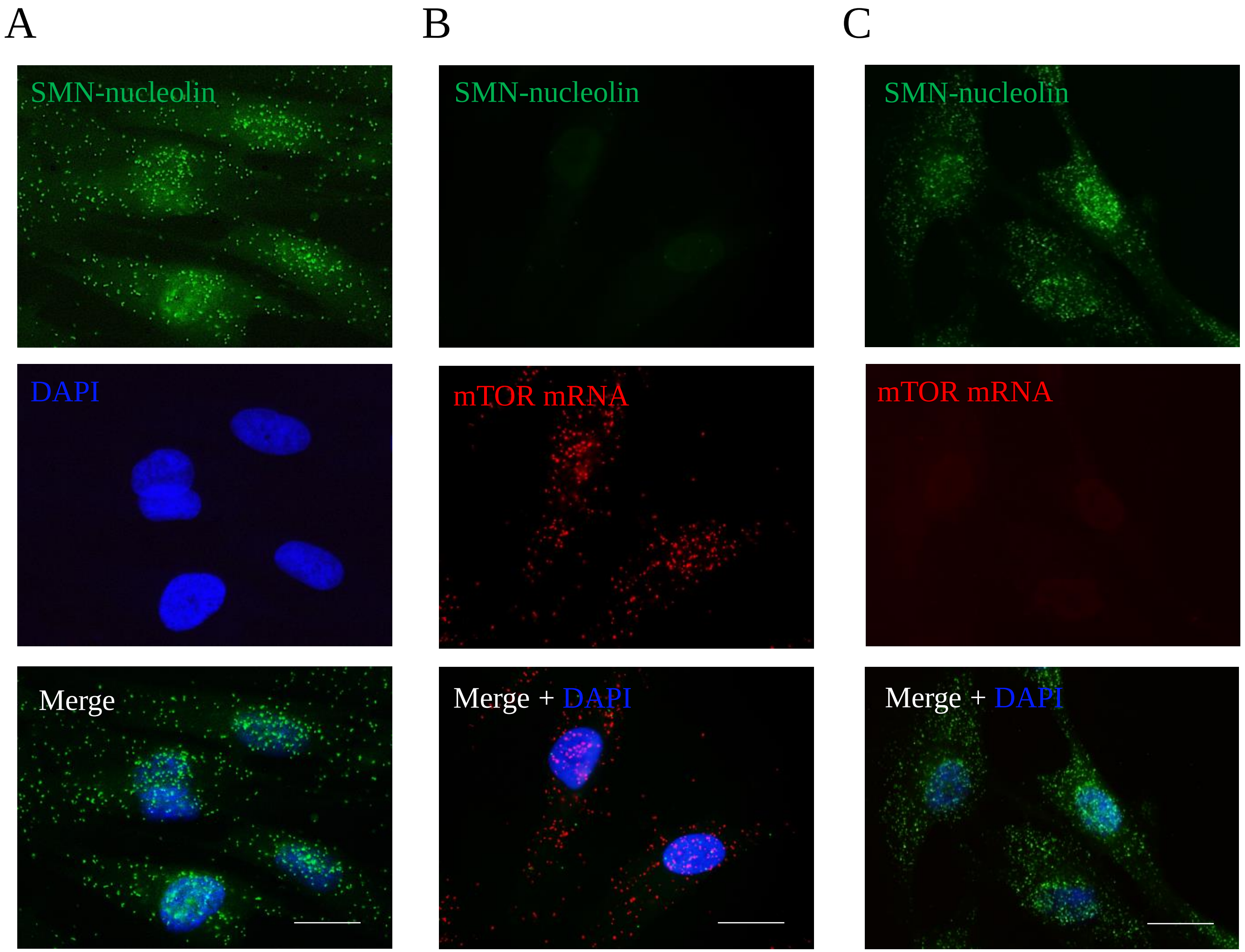

Figure S8. PlaLock controls. The specificity of PlaLock Assay was tested by a combination of appropriate controls. (**A**) The efficiency of PLA by using the Abs-plu/-minus complexes pre-formation was tested. (**B**) PLAlock step was performed omitting the SMN primary antibody. (**C**) PLALock step was performed omitting the mTOR mRNA padlock probe. Scale bar, 10 µm.

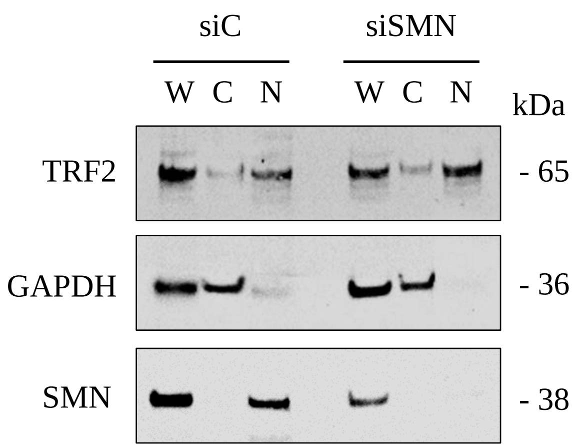

Figure S9. Subcellular fractionation controls. Immunoblotting validating the subcellular fractionation of siSMN- or siC-transfected hTert-Fibroblasts. Telomeric repeat-binding factor 2 (TRF2) and Glyceraldehyde 3-phosphate dehydrogenase (GAPDH) were detected as nuclear and cytoplasmic marker, rispectively. SMN was checked to confirm SMN silencing.

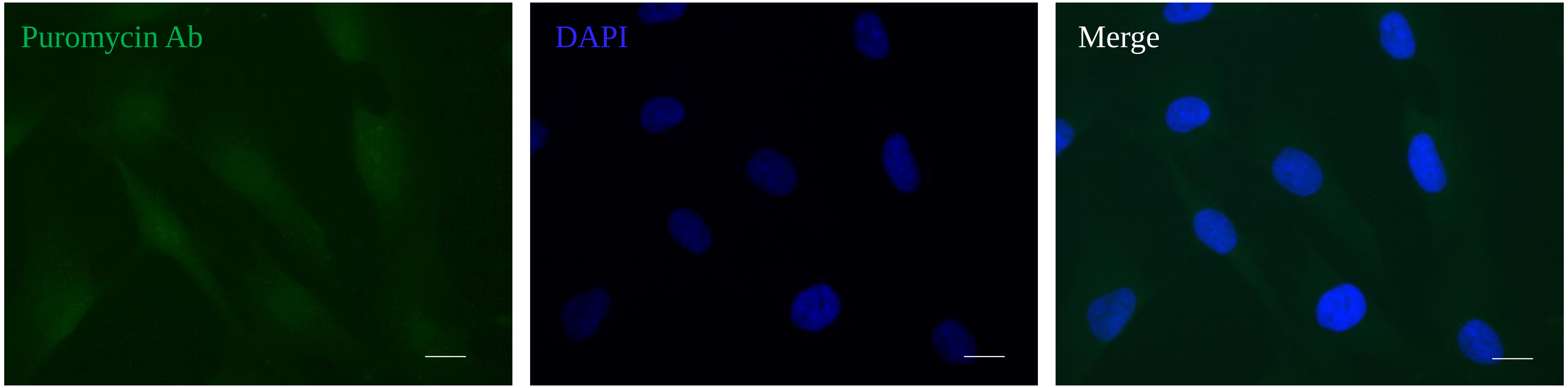

Figure S10. Puro-PLA control. Puro-PLA specificity was validated by using only the anti-puromycin antibody in the assay (Puromycin Ab). Nuclei were labelled with DAPI (blue). Scale bar, 10 μm.

Table S1. Oligos used in this study

Primer name	Primer sequence (5'-3')
RT-PCR POLB F	GTGAGACAAAGTTCATGGGTGT
RT-PCR POLB R	GTGAAACCCTTTTCTAGGGCAT
RT-PCR mTOR F	ACTGGAGGCTGATGGACACAA
RT-PCR mTOR R	CGACCAGTGAGCTTATCTCGA
Padlock Probe mTOR	CAACATTCTTGTTAGTTTTTTTTTCTCAATTCTGCTACTTTACTAC<br><br>CTCAATTCTGCTACTGTACTACTTTTTTCCTCCATAGCATTGGT
RCA Primer	AGTACAGTAGCAGAATTGAG
AlexaFluor 595-labelled probe	CTCAATTCTGCTACTTTACTAC
